# Supplementary material for: Cartography of Genomic Interactions Enables Deep Analysis of Single-Cell Expression Data
Source: Nat Commun. 2023 Feb 8;14:679. doi: 10.1038/s41467-023-36383-6 (PMC9908983; doi:10.1038/s41467-023-36383-6)
Supplement: Supplementary file 1 — Supplementary materials [file 41467_2023_36383_MOESM1_ESM.pdf]

Cartography of Genomic Interactions Enables Deep Analysis of Single-Cell Expression Data Supporting information

Islam and Xing

Contents

1 Higher-order gene-gene interactions 3

2 Information content of genomaps 3
- A Spatial entropy in 1D and 2D . . . 3
- B Mutual entropy between two random variables denoting two genes . . . 4
- C Calculation of mutual entropy for Gaussian random functions . . . 4

3 Genomaps generated from the TM dataset 6

4 3D genomaps generated from the TM dataset 11

5 Analysis of features extracted from genomaps using Xception DNN 14

6 Cell compositions of the analyzed datasets 17

7 GenoNet architecture 19

8 Analysis of interaction matrix and grid distance matrix 21

9 Analysis of genomap performance for selection of different number of HVGs and PCs 23

10 Color legends for cell classes 24

11 Reference 25

1. Higher-order gene-gene interactions

The genomap calculations presented in the main manuscript are based on two-way gene-gene interactions. The formulation can be extended to include n-way interactions among the genes. For example, for three-gene interactions, we can re-define the probability function $p(\mathbf{x})$ (1)

$$p(\mathbf{x}) = \frac{1}{Z} e^{-J(\mathbf{x})},$$

where

$$J(\mathbf{x}) = \frac{1}{2} \sum_{ij} \mathbf{x}_i \mathbf{M}_{ij} \mathbf{x}_j - \sum_i \beta_i \mathbf{x}_i - \frac{1}{6} \sum_{ijk} \mathbf{\Gamma}_{ijk} \mathbf{x}_i \mathbf{x}_j \mathbf{x}_k$$

with $\mathbf{M}$ denoting the inverse of covariance matrix $\mathbf{\Omega}$, $\boldsymbol{\beta} = \mathbf{\Omega}^{-1} \langle \boldsymbol{x} \rangle$ and

$$Z = \int d^N \mathbf{x} e^{-J(\mathbf{x})}.$$

By using standard perturbation theory, we find

$$\mathbf{\Gamma}_{ijk} = \sum_{\alpha\beta\gamma=1} \langle \mathbf{x}_\alpha \mathbf{x}_\beta \mathbf{x}_\gamma \rangle \mathbf{M}_{i\alpha} \mathbf{M}_{j\beta} \mathbf{M}_{k\gamma}.$$

The nonlinear case with the three-gene (and higher order) interactions is not amenable to exact analytic calculation and finding a closed-form solution for the gene-gene pairwise interaction strength matrix is difficult. Therefore, we use standard perturbation theory, which assumes that the characteristic three-gene (and higher order) correlation function is much smaller than the pairwise correlation. Indeed, as found in Ref. (1) for analysis of microarray data from genes in Saccharomyces cerevisiae chemostat cultures, the mean value of $\langle \mathbf{x}_i \mathbf{x}_j \mathbf{x}_k \rangle^2$ for $i \neq j, i \neq k, j \neq k$ was found to be 0.035, which is an order of magnitude smaller than $|\langle \mathbf{x}_i \mathbf{x}_j \rangle|^3$ for $i \neq j$, which was equal to 0.187. The same observation has been used in many other studies (2).

2. Information content of genomaps

For a system consisting of m cells with n genes per cell, we have n gene expression vectors $\mathbf{x}_1, \ldots, \mathbf{x}^n$. The entropy of the system can be written as (1–4)

$$H = -\sum_{\mathbf{x}} p(\mathbf{x}) \ln p(\mathbf{x}) + J \quad [1]$$

subject to the constraint

$$\sum_{\mathbf{x}} p(\mathbf{x}) = 1. \quad [2]$$

Here the first part of Eq. 1 denotes the space-independent entropy and J denotes the spatial entropy describing the mutual information among the genes (see below).

A. Spatial entropy in 1D and 2D. Following Refs. (3, 4), we divide a 2D image of width W and height H in n overlapping sub regions or windows with width of w_x and height of w_y ($w_x < W, w_y < H$). The spatial entropy is the sum of mutual entropy between the gene located at the center of the k-th window (g^k) and all other genes located in the window (g^k_{ij}). Mathematically, the spatial entropy can be written as (3, 4)

$$J_{2D} = \sum_{k=1}^{k=n} \sum_{i=1}^{i=w_x} \sum_{j=1}^{i=w_y} J_{g^k g^k_{ij}}. \quad [3]$$

A commonly used window size is 3×3 (3, 4). The spatial entropy among the genes in a 1D vector with windows of size w_x can be written as

$$J_{1D} = \sum_{k=1}^{k=n} \sum_{i=1}^{i=w_x} J_{g^k g^k_i}. \quad [4]$$

B. Mutual entropy between two random variables denoting two genes. To compute the mutual entropy between the gene g^k at the center and another gene g_{ij}^k in a window, let us assume that the expressions of the two genes are described by two discrete random variables ξ and η, respectively. The observed values of ξ and η in m cells are denoted by $\mathbf{x}_i, i = 1, \ldots, m$ and $\mathbf{y}_k, k = 1, \ldots, m$ with probabilities $P_\xi(i)$ and $P_\eta(k)$. The joint probability of ξ and η taking on the values $\mathbf{x}_i$ and $\mathbf{y}_k$, respectively, is denoted by $P_{\xi\eta}(i, k)$. According to Shannon (5), in this case the amount of information about the variable ξ contained in the variable η or the amount of information about the variable η contained in the variable ξ can be calculated by:

$$J(\xi, \eta) = -\sum_{i=1}^{m}\sum_{k=1}^{m} P_{\xi\eta}(i,k) \log \frac{P_\xi(i)P_\eta(k)}{P_{\xi\eta}(i,k)}. \quad [5]$$

When $P_{\xi\eta}(i, k) = 0$, the corresponding term in the summation is taken to be zero (5). The function defined by Eq. 5 is symmetrical in ξ and η. For continuous case where the probability distributions P_ξ, P_η and $P_{\xi\eta}$ are defined in terms of densities $p_\xi(\mathbf{x}), p_\eta(\mathbf{y})$ and $p_{\xi\eta}(\mathbf{x}, \mathbf{y})$, we can write Eq. 5 as:

$$J(\xi, \eta) = -\iint p_{\xi\eta}(\mathbf{x}, \mathbf{y}) \log \frac{p_\xi(\mathbf{x})p_\eta(\mathbf{y})}{p_{\xi\eta}(\mathbf{x}, \mathbf{y})} d\mathbf{x}d\mathbf{y}, \quad [6]$$

where $d\mathbf{x} = d\mathbf{x}_1 \cdots d\mathbf{x}_m, d\mathbf{y} = d\mathbf{y}_1 \cdots d\mathbf{y}_m$.

C. Calculation of mutual entropy for Gaussian random functions. Explicit calculation of $J(\xi, \eta)$ requires the knowledge of multi-dimensional probability distribution functions of the random variables ξ and η. Let us consider the case where the variables ξ and η are Gaussian, i.e., when all the multi-dimensional probability distributions for ξ, η and the pair (ξ, η) are multivariate normal. For simplicity, we assume that the expectations of all components of ξ and η are equal to zero (this assumption is not restrictive since the information $J(\xi, \eta)$ is independent of the values of the expectations). In this case, the probability distributions for ξ, η, and ζ will have densities of the form

$$p_\xi(\mathbf{x}) = \frac{1}{(2\pi)^{k/2}(\det \mathbf{A})^{1/2}} e^{-\frac{1}{2}(\mathbf{x}^T\mathbf{A}^{-1}\mathbf{x})}, \quad [7]$$

$$p_\eta(\mathbf{y}) = \frac{1}{(2\pi)^{l/2}(\det \mathbf{B})^{1/2}} e^{-\frac{1}{2}(\mathbf{y}^T\mathbf{B}^{-1}\mathbf{y})}, \quad [8]$$

$$p_{\xi,\eta}(\mathbf{z}) = \frac{1}{(2\pi)^{\frac{k+l}{2}}(\det \mathbf{C})^{1/2}} e^{-\frac{1}{2}(\mathbf{z}^T\mathbf{C}^{-1}\mathbf{z})}. \quad [9]$$

Here

$$\mathbf{z} = (\mathbf{x}, \mathbf{y}) = (\mathbf{x}_1, \cdots, \mathbf{x}_k, \mathbf{y}_1, \cdots, \mathbf{y}_l), \mathbf{C} = \left\| \begin{matrix} \mathbf{A} & \mathbf{D} \\ \mathbf{D}' & \mathbf{B} \end{matrix} \right\|,$$

and $\mathbf{A}, \mathbf{B}, \mathbf{D}$ are moment matrices defined as

$$\mathbf{A} = \|\mathbf{M}\xi_i\xi_j\| = \|a_{ij}\|, \quad \mathbf{B} = \|\mathbf{M}\eta_i\eta_j\| = \|b_{ij}\|, \quad \mathbf{D} = \|\mathbf{M}\xi_i\eta_j\| = \|d_{ij}\|.$$

The moment matrices can be computed as follows:

$$\begin{array}{l} \int \mathbf{x}_i\mathbf{x}_j p_\zeta(\mathbf{z})d\mathbf{z} = \int \mathbf{x}_i\mathbf{x}_j p_\xi(\mathbf{x})d\mathbf{x} = \mathbf{M}\xi_i\xi_j = a_{ij}, \\ \int \mathbf{y}_i\mathbf{y}_j p_\zeta(\mathbf{z})d\mathbf{z} = \int \mathbf{y}_i\mathbf{y}_j p_\eta(\mathbf{y})d\mathbf{y} = \mathbf{M}\eta_i\eta_j = b_{ij}, \\ \int \mathbf{x}_i\mathbf{y}_j p_\zeta(\mathbf{z})d\mathbf{z} = \mathbf{M}\xi_i\eta_j = d_{ij}. \end{array} \quad [10]$$

On substituting these expressions in Eq. 6, we have

$$J(\xi, \eta) = -\int \left\{-\frac{1}{2}\log\frac{\det \mathbf{A} \cdot \det \mathbf{B}}{\det \mathbf{C}} + \frac{1}{2}\left[\left(\mathbf{x}^T\mathbf{A}^{-1}\mathbf{x}\right) + \left(\mathbf{y}^T\mathbf{B}^{-1}\mathbf{y}\right) - \left(\mathbf{z}^T\mathbf{C}^{-1}\mathbf{z}\right)\right]\right\} p_\zeta(\mathbf{z})d\mathbf{z}. \quad [11]$$

Next, we can write:

$$\begin{array}{l} \int \left(\mathbf{x}^T\mathbf{A}^{-1}\mathbf{x}\right) p_\zeta(\mathbf{z})d\mathbf{z} = \int \left(\mathbf{x}^T\mathbf{A}^{-1}\mathbf{x}\right) p_\xi(\mathbf{x})d\mathbf{x} = \mathcal{T}\left(\mathbf{A}^{-1}\mathbf{A}\right) = m \\ \int \left(\mathbf{y}^T\mathbf{B}^{-1}\mathbf{y}\right) p_\zeta(\mathbf{z})d\mathbf{z} = \int \left(\mathbf{y}^T\mathbf{B}^{-1}\mathbf{y}\right) p_\eta(\mathbf{y})d\mathbf{y} = \mathcal{T}\left(\mathbf{B}^{-1}\mathbf{B}\right) = m \\ \int \left(\mathbf{z}^T\mathbf{C}^{-1}\mathbf{z}\right) p_\zeta(\mathbf{z})d\mathbf{z} = \mathcal{T}\left(\mathbf{C}^{-1}\mathbf{C}\right) = 2m, \end{array} \quad [12]$$

where $\mathcal{T}(\mathbf{K})$ denotes the trace of matrix $\mathbf{K}$, i.e., the sum of its diagonal elements. Therefore, we can write a simplified form of Eq. 11 as follows:

$$J(\xi, \eta) = \frac{1}{2}\log\frac{\det \mathbf{A} \cdot \det \mathbf{B}}{\det \mathbf{C}}. \quad [13]$$

When ξ and η are one-dimensional random variables (as in the case of a gene expression vector), Eq. 13 becomes ((6) p. 216)

$$J(\xi, \eta) = -\frac{1}{2} \log \left[1 - r^2(\xi, \eta)\right], \qquad [14]$$

where $r(\xi, \eta)$ is the correlation coefficient of ξ and η. The plot of the mutual entropy between two random variables (denoting two gene expression vectors) are shown in Fig. S1. Note that Eq. 14 can be extended directly to the general case of arbitrary multidimensional vectors and this has been discussed in detail in Ref. (6) page 217.

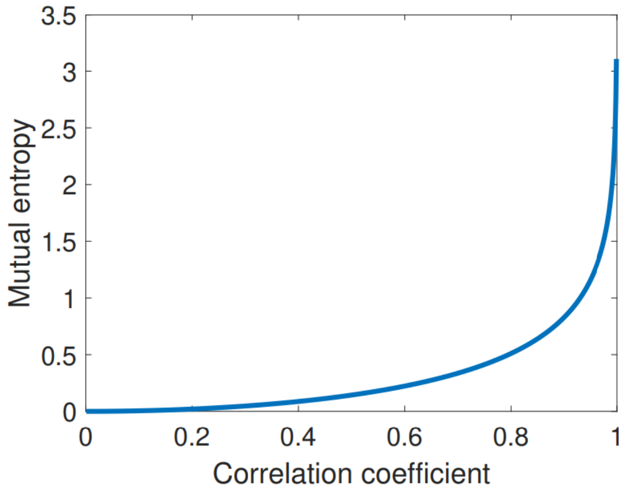

Fig. S1. Mutual entropy plotted against correlation coefficient between two genes. Source data are provided as a Source Data file.

The general formulation Eq. 1 reveals that genomap has higher entropy than other data format (such as 1D, random mapping, and vec2image (7)). The space-independent part of the entropy equation is same for all the data formats. However, genomaps have higher spatial entropy than other data formats because of the higher spatial correlation among the neighborhood genes. For random mapping, the spatial entropy is lower because of the low correlation among the neighborhood genes. The same argument is applicable for 1D expression vectors. In vec2image, t-SNE or other embedding method is used to create the images without any constrain(s) on the spatial locations of the genes. In other words, similar genes get clustered without any optimization of their positions for maximizing the entropy. As a result, many genes located at the outer region of the clusters have less number of neighbors than those located in the center of the cluster. The same is true for those non-clustered (i.e. isolated) genes. Thus the entropy of vec2image images is less than that of genomaps. To be more specific, in vec2image, the grid size is normally taken much larger than the gene number (as an example, in study of Ref. (7), the authors have used 120X120=14400 grid points for < 5000 genes (see Fig. 3 of Ref. (7)). For such sparse gene positions, the spatial entropies of the genes located at the outer region of clusters and those isolated genes are very small due to the reduced numbers of neighboring genes. There are hundreds of such peripheral and isolated genes in vec2image images as seen in all four plots of Fig. 3E of Ref. (7)). The computed spatial entropy for four different data formats (1D, 2D random, genomap, and vec2image) for three scRNA-seq datasets are reported in Table S1, where it is seen that genomap achieves much higher entropy than all other data formats.

Table S1. Spatial entropy for different methods

Dataset	1D gene expression	Random map	Vec2image	Genomap
T cell landscape	5.0097	5.1222	4.3703	9.9993
Retinal bipolar neurons	1.9993	1.5951	1.7217	9.1272
Ischaemic sensitivity-lung	5.2192	21.0778	22.3455	28.5176

3. Genomaps generated from the TM dataset

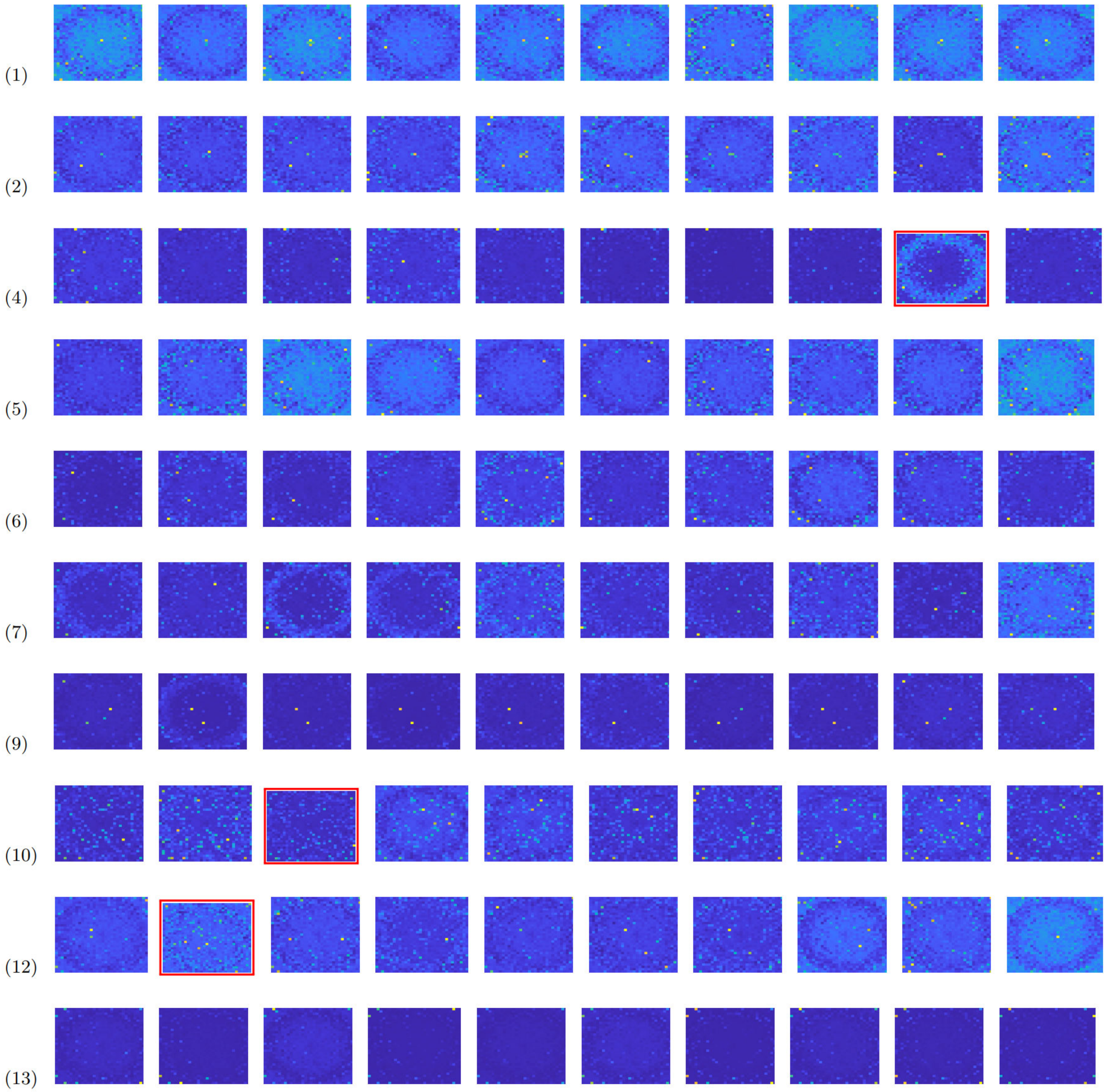

Fig. S2. Genomaps of 45 different cell classes in the TM data. Genomaps of other 10 classes in the TM data are shown in Fig. 2 of the manuscript. Cell classes corresponding to the label numbers on the left are shown in Fig. S3. Misclassified genomaps by the proposed technique are labeled by red box.

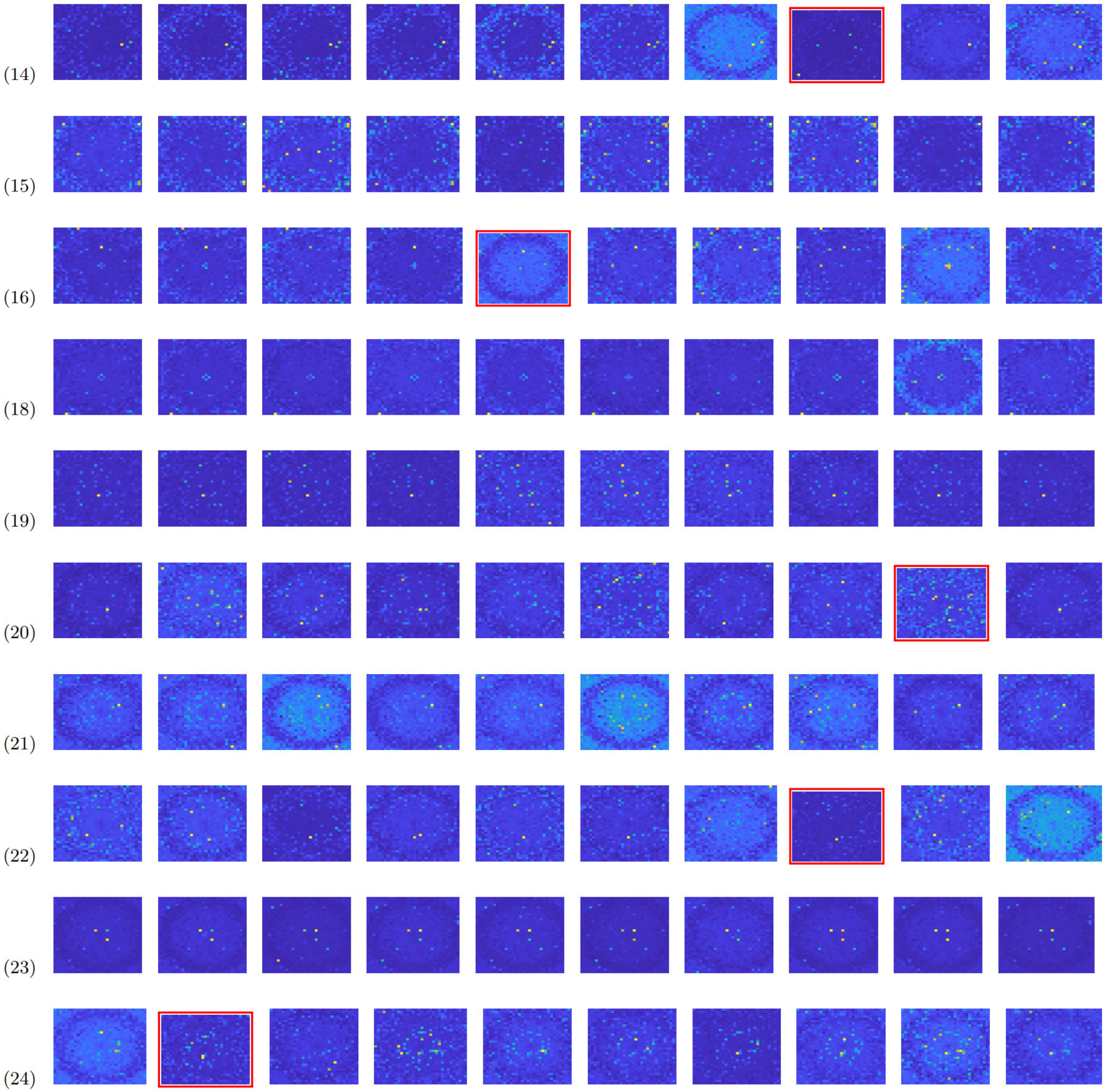

Fig. S2 (Cont.)

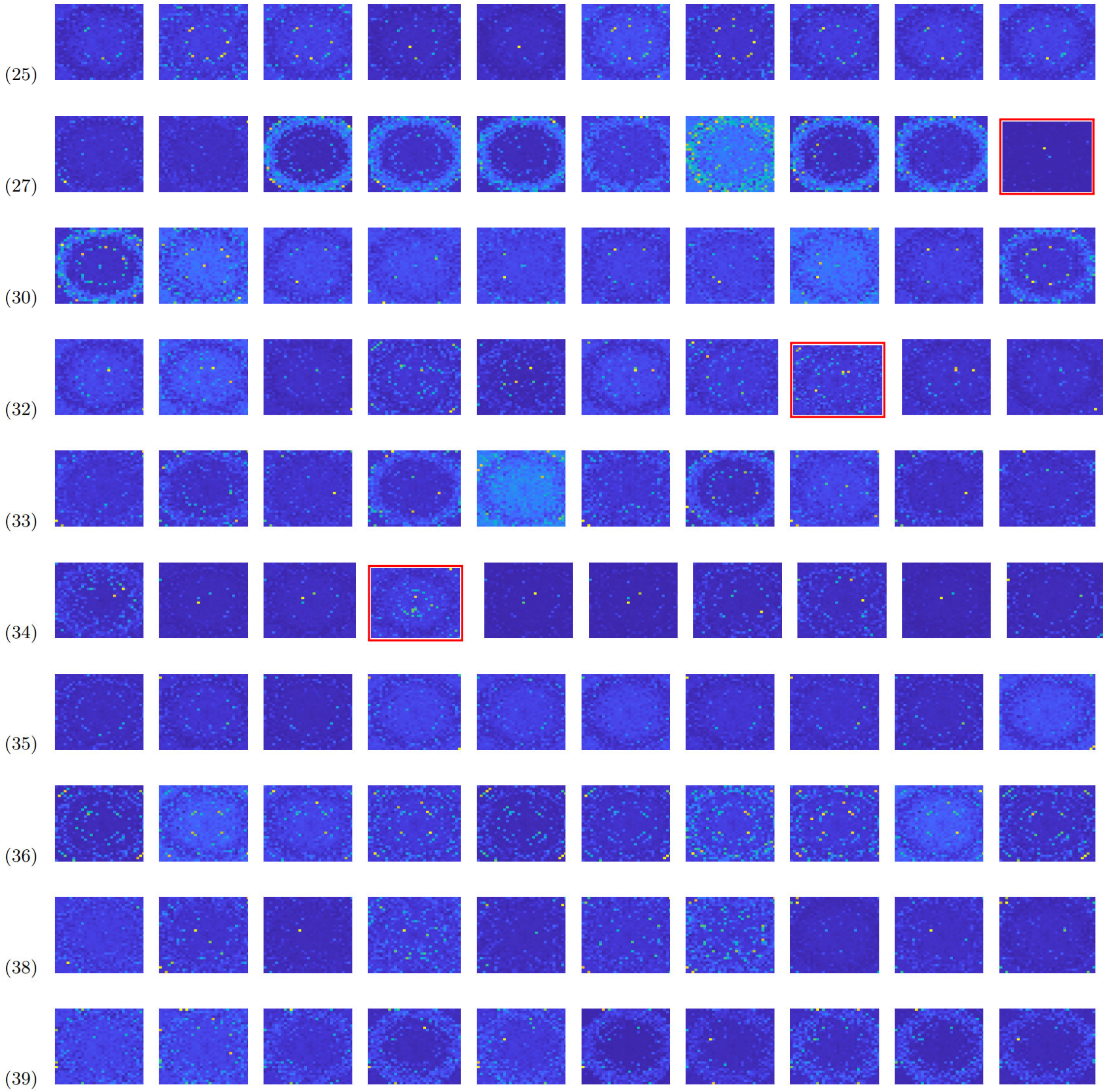

Fig. S2 (Cont.)

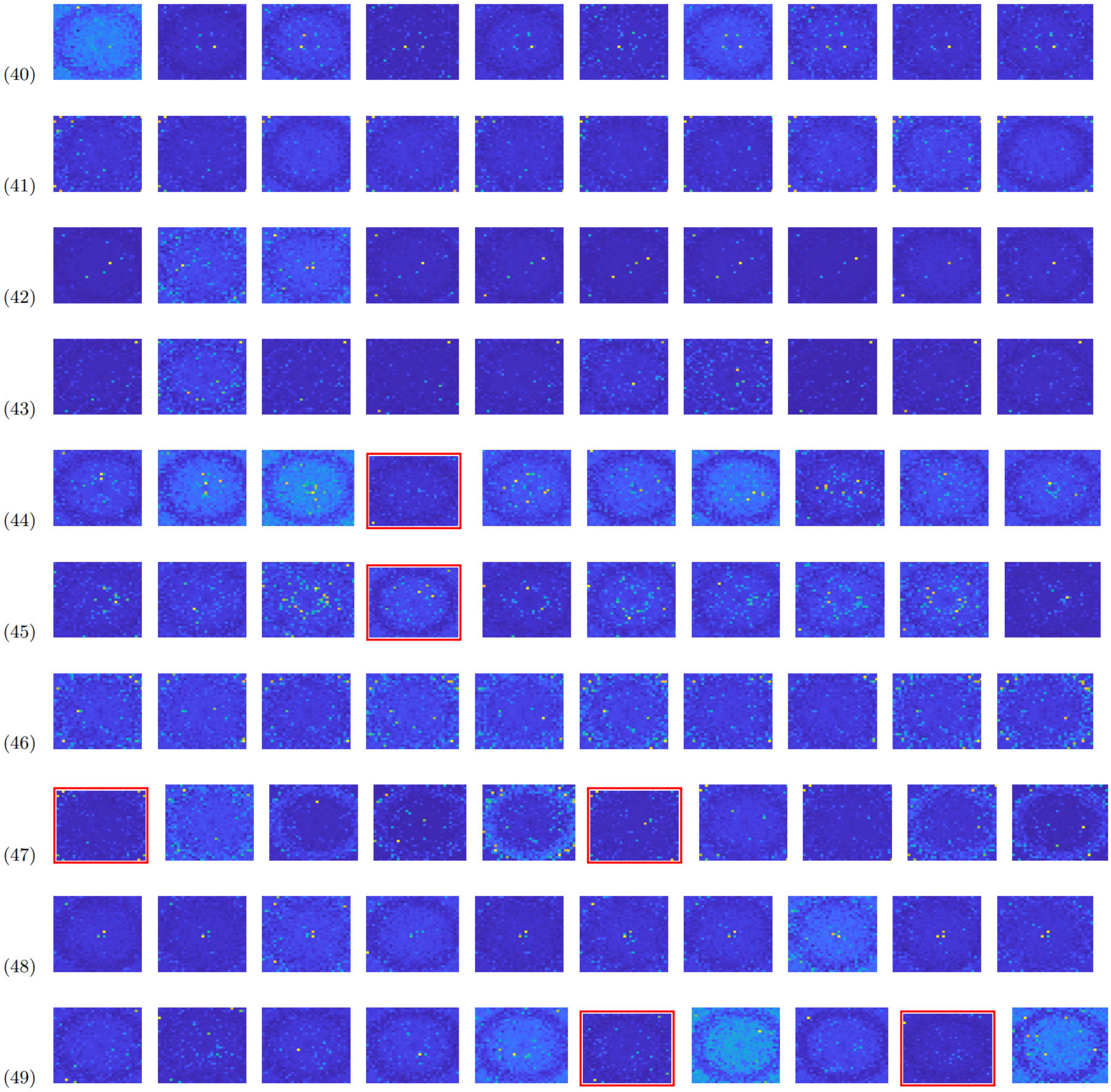

Fig. S2 (Cont.)

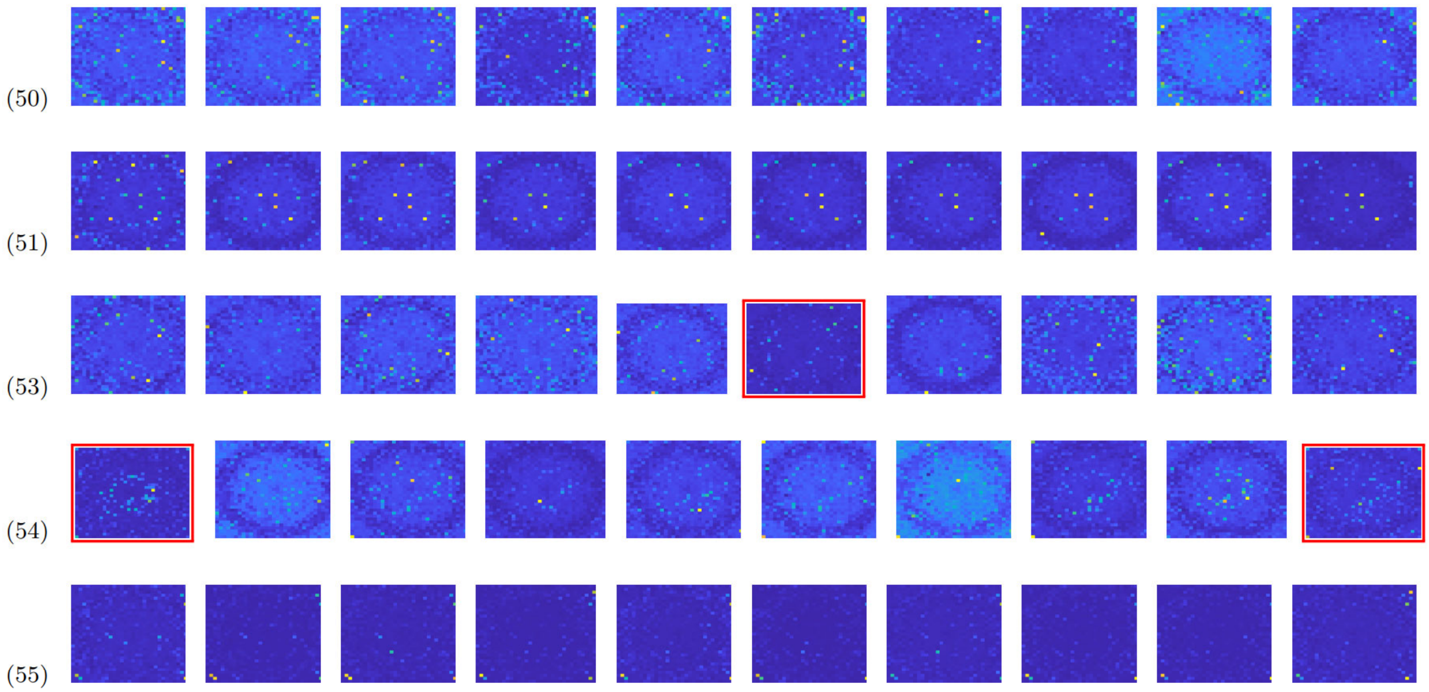

Fig. S2 (Cont.)

No	Cell class	Figure
1	B cell	Fig.S2
2	DN1 thymic pro-T cell	Fig.S2
3	Fraction A pre-pro B cell	Fig.2
4	Langerhans cell	Fig.S2
5	T cell	Fig.S2
6	alveolar macrophage	Fig.S2
7	basal cell	Fig.S2
8	basal cell of epidermis	Fig.2
9	basophil	Fig.S2
10	bladder cell	Fig.S2
11	bladder urothelial cell	Fig.2
12	blood cell	Fig.S2
13	cardiac muscle cell	Fig.S2
14	ciliated columnar cell of tracheobronchial tree	Fig.S2
15	classical monocyte	Fig.S2
16	dendritic cell	Fig.S2
17	duct epithelial cell	Fig.S2
18	early pro-B cell	Fig.S2
19	endocardial cell	Fig.S2
20	endothelial cell	Fig.S2
21	endothelial cell of hepatic sinusoid	Fig.S2
22	epithelial cell	Fig.S2
23	erythroblast	Fig.S2
24	fibroblast	Fig.S2
25	granulocyte	Fig.S2
26	granulocytopoietic cell	Fig.2
27	hematopoietic precursor cell	Fig.S2
28	hepatocyte	Fig.2
29	immature B cell	Fig.2
30	immature T cell	Fig.S2
31	keratinocyte	Fig.2
32	kidney capillary endothelial cell	Fig.S2
33	kidney cell	Fig.S2
34	kidney collecting duct epithelial cell	Fig.S2
35	kidney loop of Henle ascending limb epithelial cell	Fig.S2
36	kidney proximal straight tubule epithelial cell	Fig.S2
37	late pro-B cell	Fig.2
38	leukocyte	Fig.S2
39	luminal epithelial cell of mammary gland	Fig.S2
40	lung endothelial cell	Fig.S2
41	macrophage	Fig.S2
42	mast cell	Fig.S2
43	mesangial cell	Fig.S2
44	mesenchymal cell	Fig.S2
45	mesenchymal stem cell	Fig.S2
46	monocyte	Fig.S2
47	myeloid cell	Fig.S2
48	natural killer cell	Fig.S2
49	neuroendocrine cell	Fig.S2
50	non-classical monocyte	Fig.S2
51	proerythroblast	Fig.S2
52	promonocyte	Fig.2
53	skeletal muscle satellite cell	Fig.S2
54	stromal cell	Fig.S2
55	type II pneumocyte	Fig.S2

Fig. S3. Cell classes in TM data

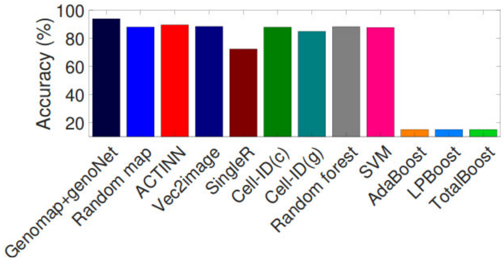

Fig. S4. Classification accuracy of the proposed genomap approach and other techniques for TM data. Source data are provided as a Source Data file.

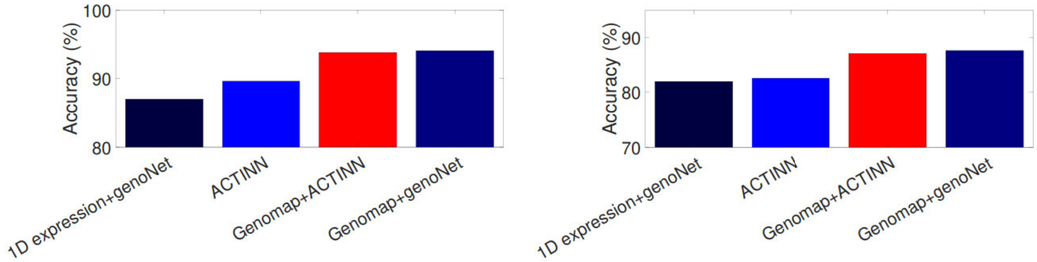

Fig. S5. Classification accuracy of the proposed genomap approach and other techniques for TM data (left) and T-cell landscape data (right). Source data are provided as a Source Data file.

4. 3D genomaps generated from the TM dataset

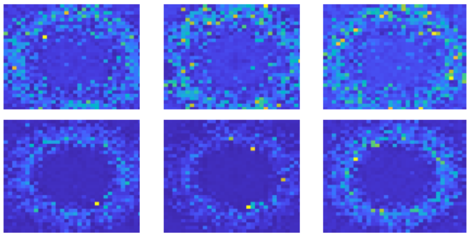

Fig. S6. 3D Genomaps of keratinocyte (1st row) and basal cell of epidermis (2nd row) at 3 different directions (from left to right).

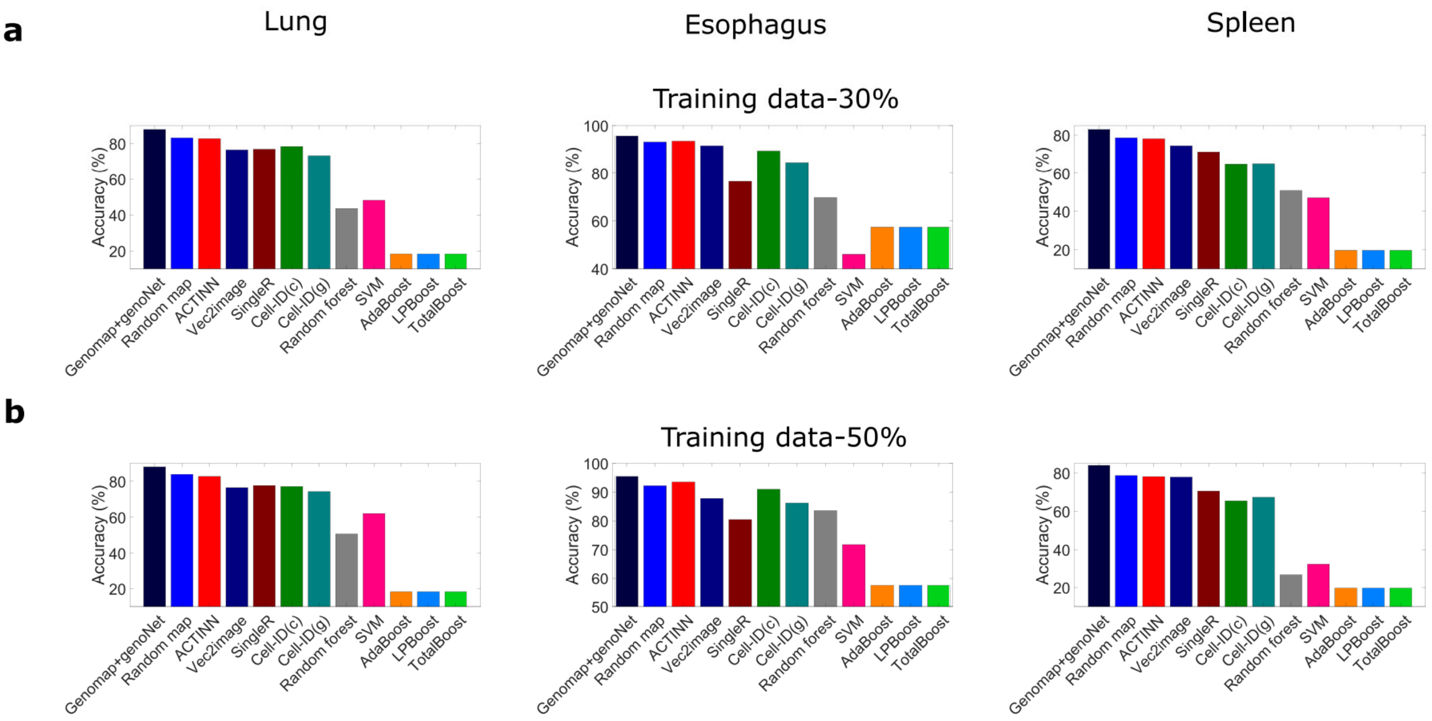

Fig. S7. Classification accuracy of ischaemic sensitivity dataset when the training data is (a) 30% and (b) 50% of the total data. Source data are provided as a Source Data file.

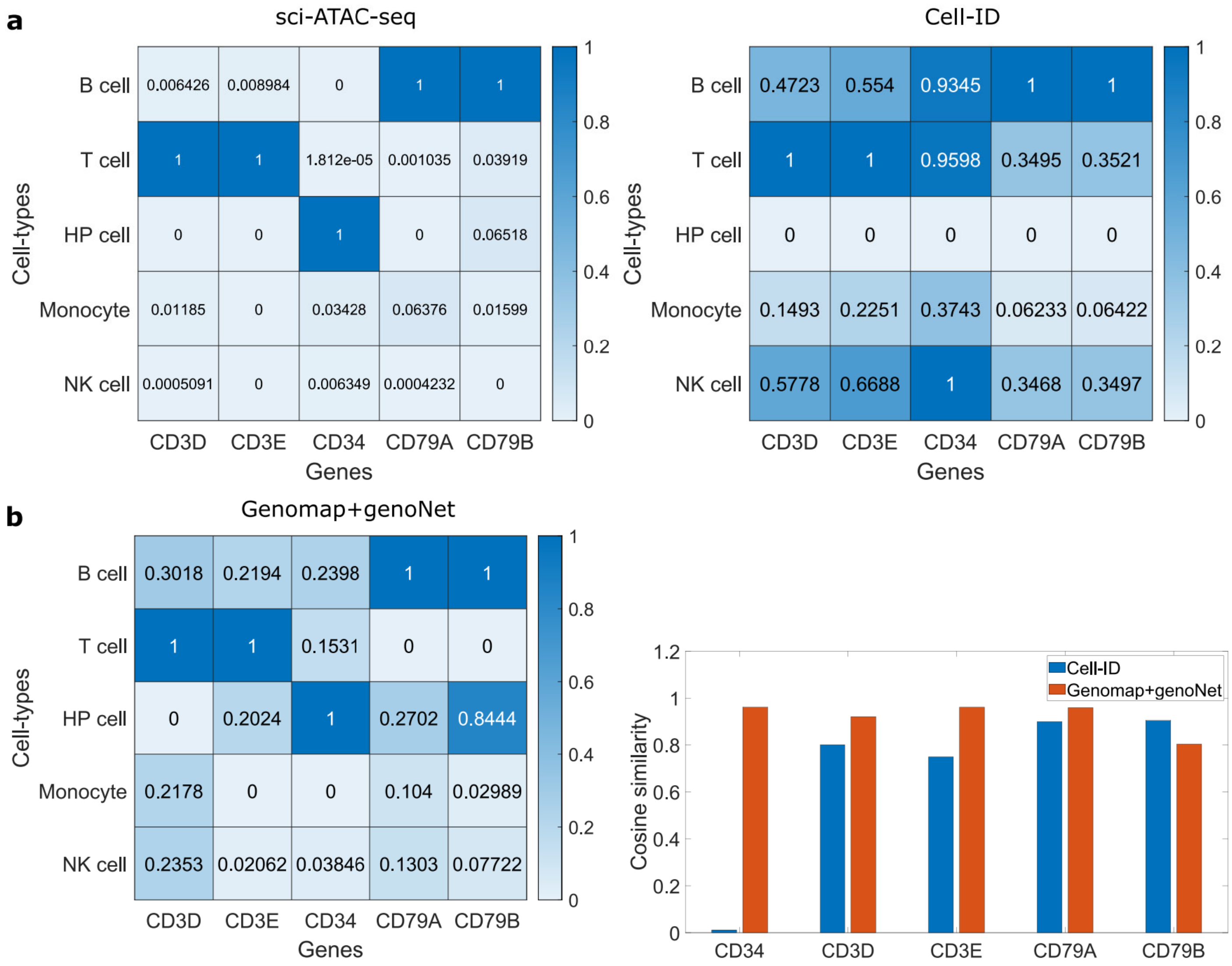

Fig. S8. Quantitative comparison of the proposed approach in discovering class-specific genes with the state-of-the-art Cell-ID technique. (a-left) The ground truth activity scores of the 5 marker genes of B-cells, T-cells, and hematopoietic (HP) cells from sci-ATAC-seq dataset. (a-right) Activity scores of the 5 genes from the Cell-ID technique. (b-left) Activity scores of the 5 genes from the proposed approach. (b-right) Cosine similarity between the ground truth and results from Cell-ID and the proposed technique. Source data are provided as a Source Data file.

5. Analysis of features extracted from genomaps using Xception DNN

To extract deep configurational features, the genomaps for all the 55 classes of the TM data are used to train an Xception deep neural network (DNN). For the cells shown in the first column of Fig. 2, some extracted features at the end of model training are are displayed in Fig. S9. It is seen that the early layers of the network are focused on local (or low level) features of genomaps whereas the deep layers extract global (or high level) features. For example, for keratinocyte cells (1st row of Fig. S9), the low level features of the doughnut are extracted by the early layers. The high level features captured by the deep layers are shown in the last four columns of Fig. S9. Different from keratinocyte cells, there are little important global features in immature B-cells (last row of Fig. S9) as most pixels other than the central ones have very low expression values (last row of Fig. 2). Indeed, the last five columns corresponding to the immature-B cells have essentially no features. Similar discussion also applies to other cell classes in Fig. S9.

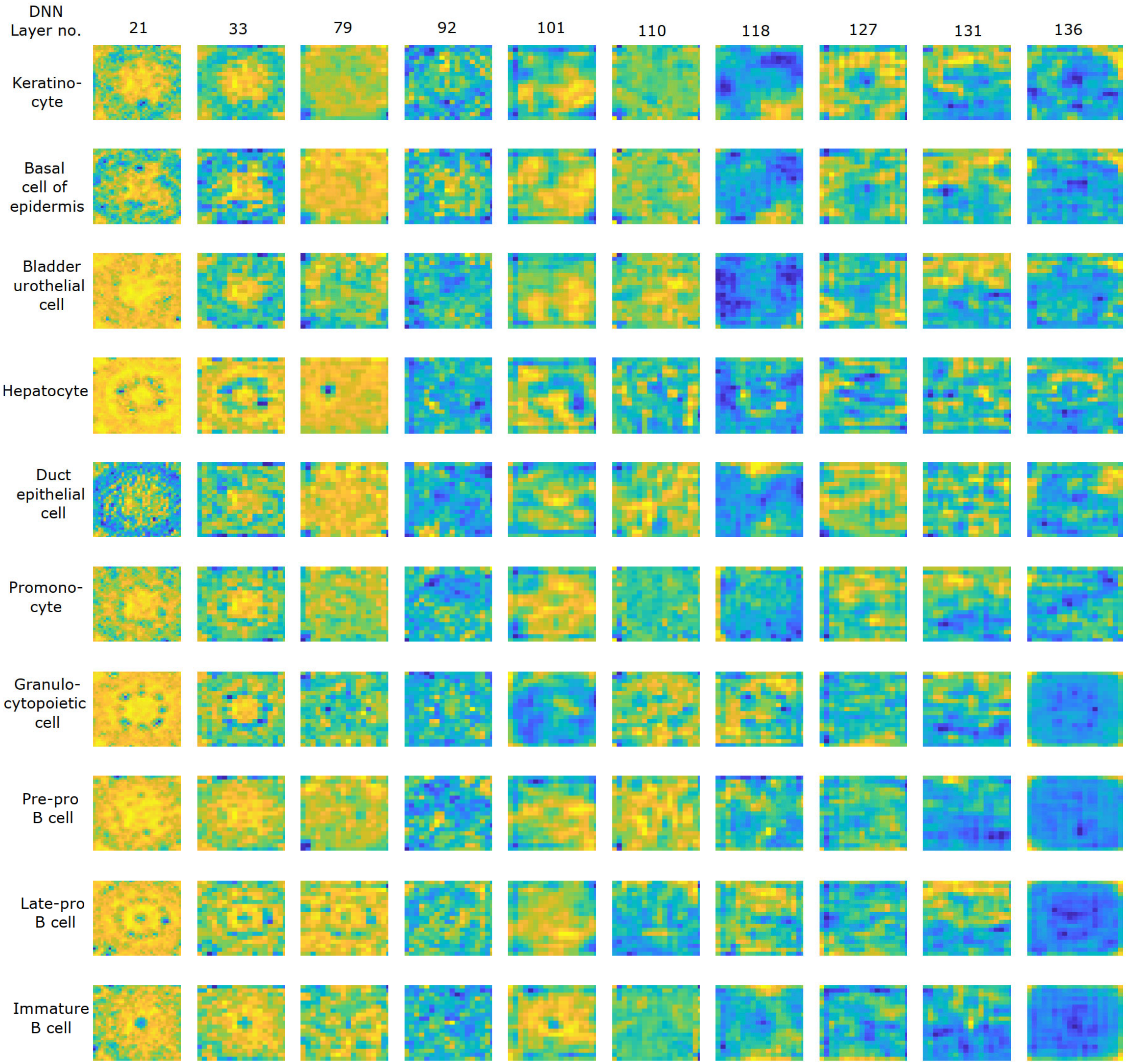

Fig. S9. Exemplary Xception network features extracted from the genomaps of 10 different cells belonging to different classes (each row corresponds to a class) from Tabula Muris dataset. Specifically, two feature maps of the cells obtained by using two different convolutional filters at layers 21, 33, 79, 92, and 110 are shown in the 1st and 2nd, 3rd and 4th, 5th and 6th, 7th and 8th, and the 9th and 10th columns, respectively. Here, the lowest and highest values in the genomaps are denoted by blue and yellow colors, respectively. It is interesting that the early layers extract features that are local (or low level) and the deep layers extract global features (or high level features). For example, in the 1st row (granulocytopoietic cells), the early layers of the network (21, 33) extract the center and circular features from the genomap. Similarly, in the early layers of the 2nd row (erythroblast cells), the features in the central region show high values. When going deeper in the network, one sees that there are no important global features in erythroblast cells (5th-10th columns). This is reasonable as most of the genes in this class have zero expression on the genomaps other than the central ones (see Fig. 2). In granulocytopoietic cells, we see that the deeper layers find some global/high level structure information in the genomaps (9th and 10th columns). This is also sensible as the genes all over the genomaps in granulocytopoietic cells have high expression values and interact each other strongly.

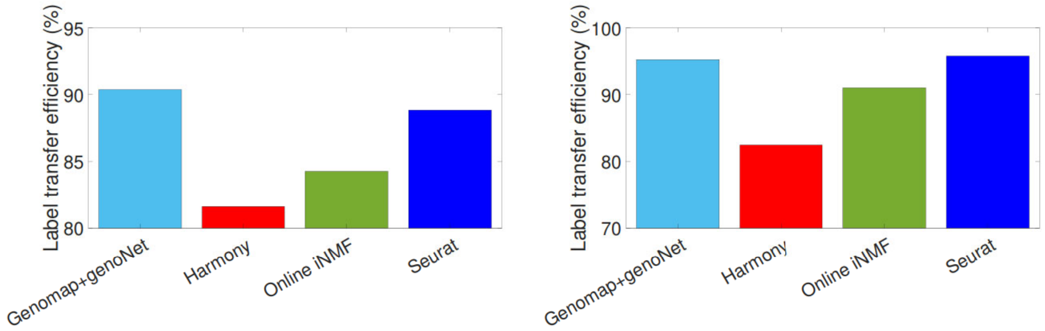

Fig. S10. Classification accuracy of the proposed approach and other techniques for pancreatic datasets by Wang et al. (left) and Xin et al (right). Source data are provided as a Source Data file.

6. Cell compositions of the analyzed datasets

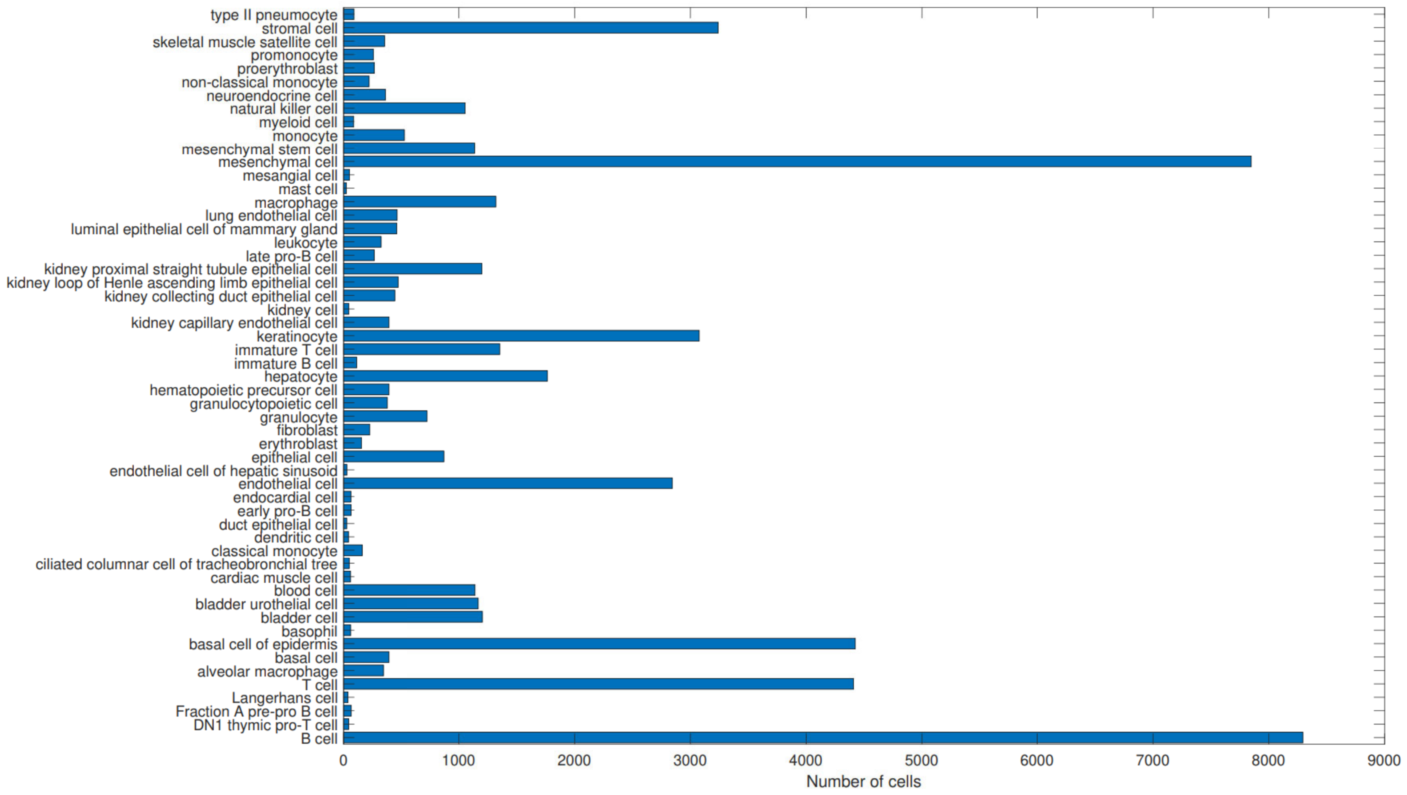

Fig. S11. Cell composition of TM dataset. Source data are provided as a Source Data file.

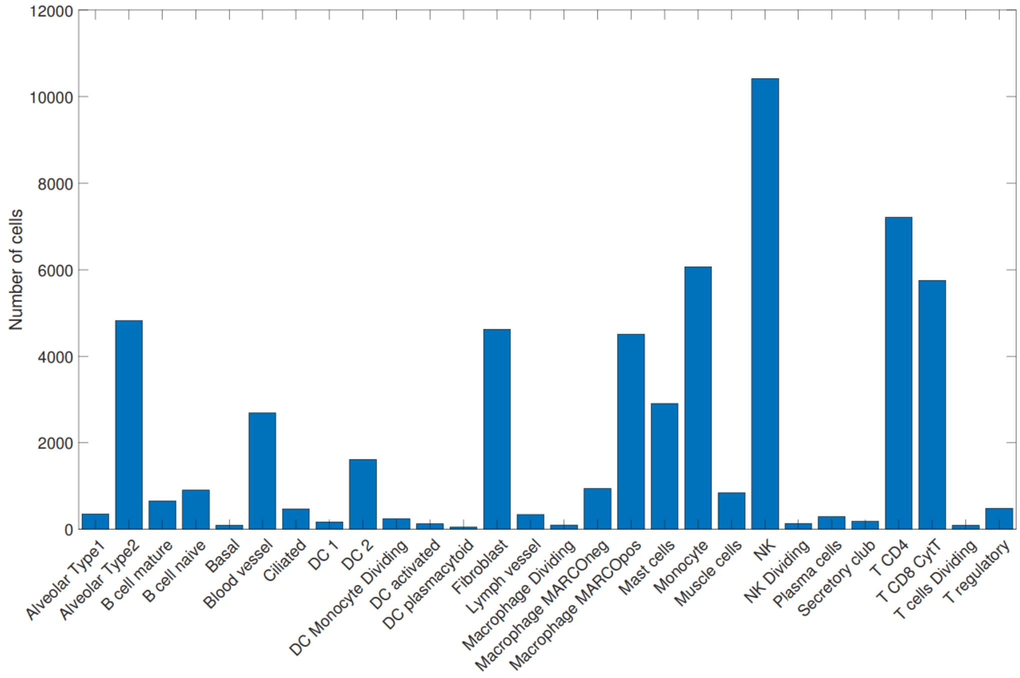

Fig. S12. Cell composition of ischaemic sensitivity dataset from lung. Source data are provided as a Source Data file.

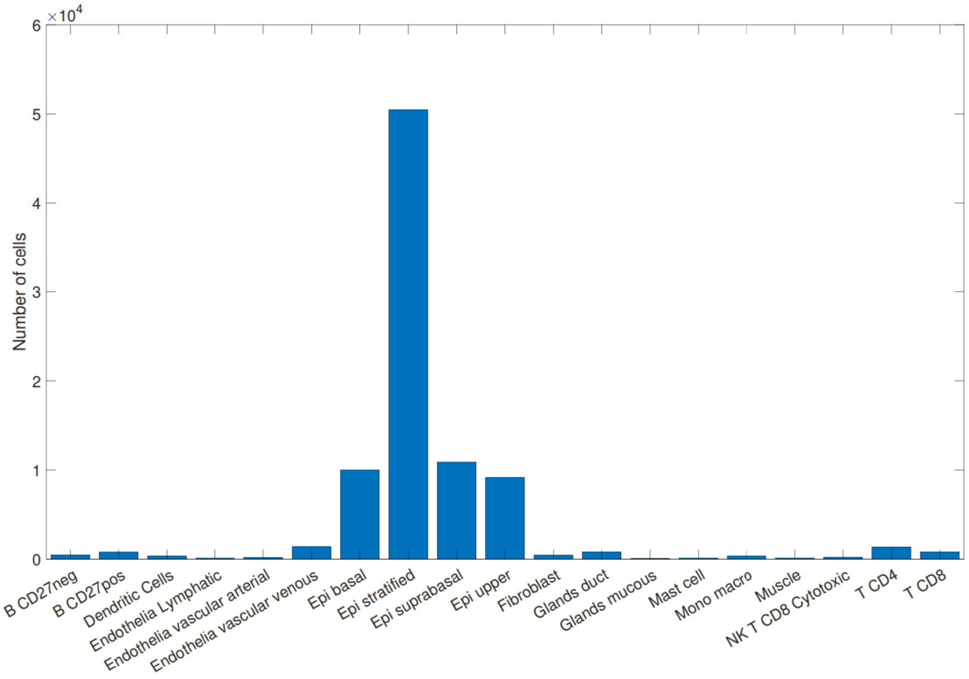

Fig. S13. Cell composition of ischaemic sensitivity dataset from esophagus. Source data are provided as a Source Data file.

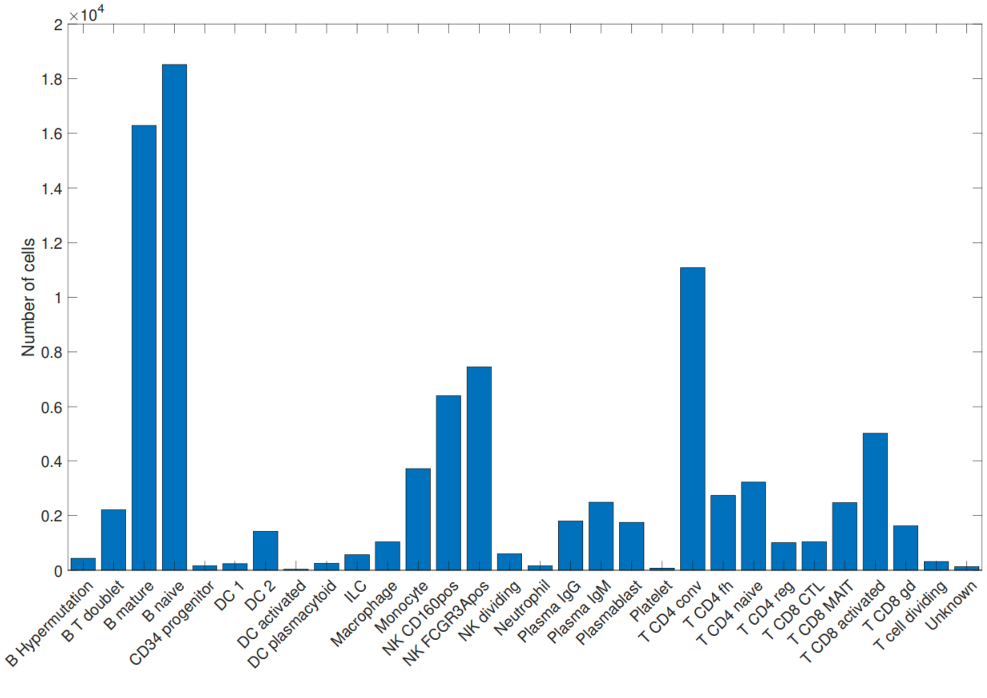

Fig. S14. Cell composition of ischaemic sensitivity dataset from spleen. Source data are provided as a Source Data file.

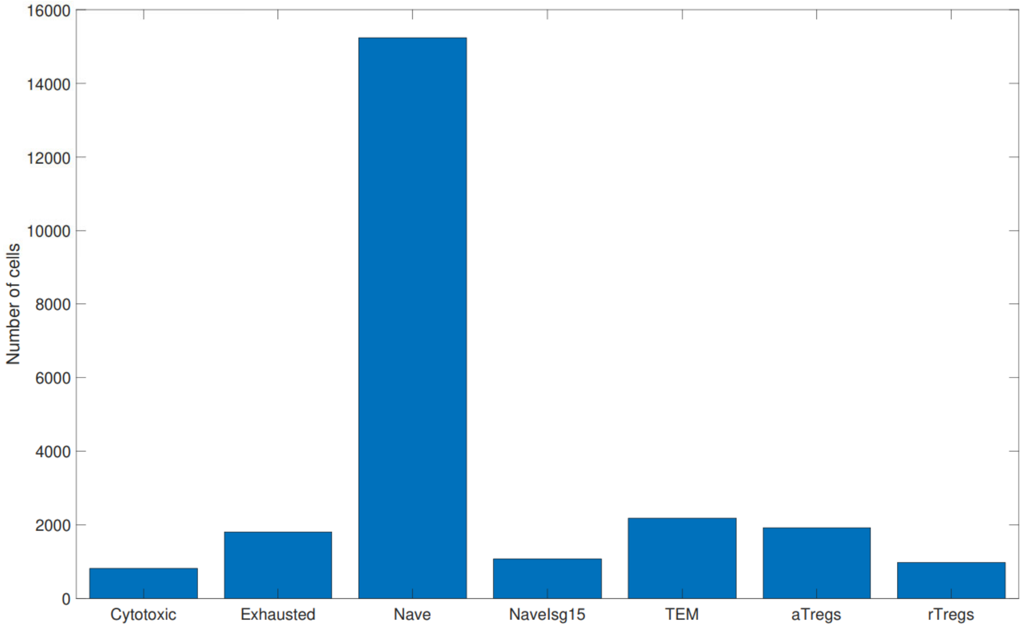

Fig. S15. Cell composition of T cell landscape dataset. Source data are provided as a Source Data file.

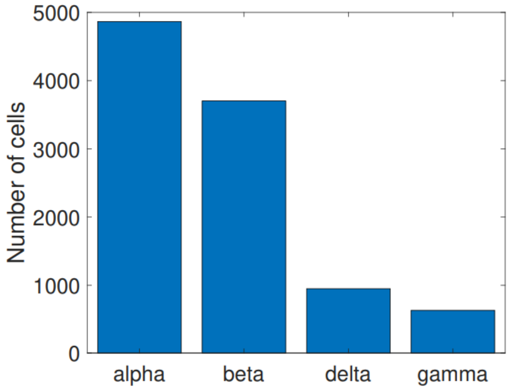

Fig. S16. Cell composition of pancreatic dataset. Source data are provided as a Source Data file.

7. GenoNet architecture

Table S2. Layer configurations of genoNet

Layer type	Activations	Learnables
Image Input	$51 \times 51 \times 1$	–
Convolution	$51 \times 51 \times 8$	Weights $3 \times 3 \times 1 \times 8$, Bias $1 \times 1 \times 8$
ReLU	$51 \times 51 \times 8$	–
Fully Connected	$1 \times 1 \times 8712$	Weights 8712×8712, Bias 8712×1
ReLU	$1 \times 1 \times 8712$	–
Fully Connected	$1 \times 1 \times 100$	Weights 100×8712, Bias 100×1
Dropout	$1 \times 1 \times 100$	–
Fully Connected	$1 \times 1 \times 20$	Weights 20×100, Bias 20×1
log softmax	$1 \times 1 \times 20$	-
Classification Output	–	-

Table S3. Performance of genomap+genoNet for different shapes of genomaps of comprehensive classification of retinal data. 1024 most highly variable genes are used to create square shaped genomaps below (case 1 and 3). 1088 most variable genes are used in creating rectangular shaped genomaps (case 2).

Shape	Cell classification accuracy
Square (32×32)	96.67%
Rectangular (64×16)	96.92%
Square with space filled with zeros (33×33)	96.68%

Table S4. Performance of genomap+genoNet for different number of selected genes from comprehensive classification of retinal data.

Number of selected genes	Cell classification accuracy
900	96.23%
1600	97.08%
2500	97.21%

Table S5. Performance of genomap+genoNet for different number of principal components from comprehensive classification of retinal data.

Number of principal components	Cell classification accuracy
900	97.59%
1600	97.38%
2500	97.67%

8. Analysis of interaction matrix and grid distance matrix

	1	2	3	4	5	6	7	8	9	10	11	12	13	14	15	16	17	18	19	20	21	22	23	24
1	0	0.9791	0.9769	1.0082	1.0014	1.0136	1.0513	1.0012	1.0047	1.0239	0.9833	1.0158	1.0332	1.0206	0.9838	1.0030	1.0149	1.0196	1.0265	1.0435	1.0424	1.0364	1.0106	1.0290
2	0.9791	0.0000	0.0076	1.0131	1.0021	1.0218	1.0676	1.0019	1.0076	1.0384	0.0649	1.0254	1.0437	1.0331	0.0637	1.0049	1.0239	0.5937	1.0428	1.0516	1.0415	1.0515	1.0199	1.0254
3	0.9769	0.0076	0.0000	1.0130	1.0020	1.0216	1.0678	1.0019	1.0075	1.0379	0.0708	1.0251	1.0451	1.0327	0.0640	1.0048	1.0237	0.6016	1.0422	1.0531	1.0444	1.0526	1.0197	1.0287
4	1.0082	1.0131	1.0130	0.0000	1.0014	0.5846	0.9912	1.0014	0.9902	0.5478	1.0108	1.0182	0.6321	0.9926	1.0109	1.0036	0.8454	1.0054	1.0306	0.6800	0.6806	0.6303	0.9431	0.7186
5	1.0014	1.0021	1.0020	1.0014	0.0000	1.0028	0.9822	1.0000	1.0010	1.0049	1.0019	1.0011	1.0004	1.0042	1.0019	0.9973	1.0029	0.9770	0.9991	0.9942	0.9967	0.9934	1.0004	0.9848
6	1.0136	1.0218	1.0216	0.5846	1.0028	0.0000	0.9758	1.0023	0.4484	0.2296	1.0179	1.0302	0.3237	0.8600	1.0181	1.0059	0.6717	1.0422	1.0508	0.4650	0.4703	0.3671	0.9521	0.5238
7	1.0513	1.0676	1.0678	0.9912	0.9822	0.9758	0	1.0092	0.9759	1.0148	1.0526	0.9095	0.9352	1.0525	1.0563	0.9630	0.9915	0.8779	0.8394	0.9101	0.8664	0.8377	0.8308	0.7782
8	1.0012	1.0019	1.0019	1.0014	1.0000	1.0023	1.0092	0.0000	1.0008	1.0040	1.0016	1.0009	1.0058	1.0035	1.0016	1.0005	1.0025	1.0065	0.9946	1.0073	1.0072	1.0054	1.0021	1.0055
9	1.0047	1.0076	1.0075	0.9902	1.0010	0.4484	0.9759	1.0008	0.0000	0.5637	1.0062	1.0105	0.7523	0.9342	1.0063	1.0021	0.9050	1.0317	1.0177	0.8347	0.8312	0.7748	0.9691	0.8340
10	1.0239	1.0384	1.0379	0.5478	1.0049	0.2296	1.0148	1.0040	0.5637	0.0000	1.0314	1.0531	0.2357	0.4756	1.0317	1.0104	0.3508	1.0648	1.0893	0.3417	0.3974	0.3634	0.9402	0.5077
11	0.9833	0.0649	0.0708	1.0108	1.0019	1.0179	1.0526	1.0016	1.0062	1.0314	0	1.0208	1.0327	1.0271	0.0618	1.0041	1.0196	0.6172	1.0350	1.0375	1.0281	1.0392	1.0159	1.0144
12	1.0158	1.0254	1.0251	1.0182	1.0011	1.0302	0.9095	1.0009	1.0105	1.0531	1.0208	0	1.0543	1.0458	1.0210	1.0069	1.0331	1.0988	0.7085	1.0493	1.0442	1.0153	1.0292	1.0237
13	1.0332	1.0437	1.0451	0.6321	1.0004	0.3237	0.9352	1.0058	0.7523	0.2357	1.0327	1.0543	0	0.6349	1.0384	1.0134	0.5642	0.8481	1.1233	0.0572	0.0631	0.0755	0.9455	0.1725
14	1.0206	1.0331	1.0327	0.9926	1.0042	0.8600	1.0525	1.0035	0.9342	0.4756	1.0271	1.0458	0.6349	0	1.0274	1.0090	0.4284	1.0581	1.0767	0.6377	0.7316	0.7925	0.9853	0.8273
15	0.9838	0.0637	0.0640	1.0109	1.0019	1.0181	1.0563	1.0016	1.0063	1.0317	0.0618	1.0210	1.0384	1.0274	0	1.0041	1.0198	0.6301	1.0354	1.0452	1.0382	1.0456	1.0166	1.0244
16	1.0030	1.0049	1.0048	1.0036	0.9973	1.0059	0.9630	1.0005	1.0021	1.0104	1.0041	1.0069	1.0134	1.0090	1.0041	0.0000	1.0065	0.9591	1.0064	1.0069	1.0122	1.0087	1.0031	0.9955
17	1.0149	1.0239	1.0237	0.8454	1.0029	0.6717	0.9915	1.0025	0.9050	0.3508	1.0196	1.0331	0.5642	0.4284	1.0198	1.0065	0	1.0492	1.0558	0.6477	0.6957	0.6684	0.9366	0.7105
18	1.0196	0.5937	0.6016	1.0054	0.9770	1.0422	0.8779	1.0065	1.0317	1.0648	0.6172	1.0988	0.8481	1.0581	0.6301	0.9591	1.0492	0	1.1307	0.7844	0.7383	0.7785	0.9871	0.6639
19	1.0265	1.0428	1.0422	1.0306	0.9991	1.0508	0.8394	0.9946	1.0177	1.0893	1.0350	0.7085	1.1233	1.0767	1.0354	1.0064	1.0558	1.1307	0.0000	1.1322	1.1253	1.0927	1.0373	1.1068
20	1.0435	1.0516	1.0531	0.6800	0.9942	0.4650	0.9101	1.0073	0.8347	0.3417	1.0375	1.0493	0.0572	0.6377	1.0452	1.0069	0.6477	0.7844	1.1322	0	0.0539	0.0949	0.9289	0.1549
21	1.0424	1.0415	1.0444	0.6806	0.9967	0.4703	0.8664	1.0072	0.8312	0.3974	1.0281	1.0442	0.0631	0.7316	1.0382	1.0122	0.6957	0.7383	1.1253	0.0539	0.0000	0.0523	0.9332	0.0948
22	1.0364	1.0515	1.0526	0.6303	0.9934	0.3671	0.8377	1.0054	0.7748	0.3634	1.0392	1.0153	0.0755	0.7925	1.0456	1.0087	0.6684	0.7785	1.0927	0.0949	0.0523	0	0.9077	0.0793
23	1.0106	1.0199	1.0197	0.9431	1.0004	0.9521	0.8308	1.0021	0.9691	0.9402	1.0159	1.0292	0.9455	0.9853	1.0166	1.0031	0.9366	0.9871	1.0373	0.9289	0.9332	0.9077	0.0000	0.8821
24	1.0290	1.0254	1.0287	0.7186	0.9848	0.5238	0.7782	1.0055	0.8340	0.5077	1.0144	1.0237	0.1725	0.8273	1.0244	0.9955	0.7105	0.6639	1.1068	0.1549	0.0948	0.0793	0.8821	0.0000

Fig. S17. The values of the first 24×24 elements in C=1-interaction matrix.

	1	2	3	4	5	6	7	8	9	10	11	12	13	14	15	16	17	18	19	20	21	22	23	24
1	0	1.0000	1.0000	1.0000	1.0000	1.4142	1.4142	1.4142	1.4142	2.0000	2.0000	2.0000	2.0000	2.2361	2.2361	2.2361	2.2361	2.2361	2.2361	2.2361	2.2361	2.8284	2.8284	2.8284
2	1.0000	0	0	0	0	0.4142	0.4142	0.4142	0.4142	1.0000	1.0000	1.0000	1.0000	1.2361	1.2361	1.2361	1.2361	1.2361	1.2361	1.2361	1.2361	1.8284	1.8284	1.8284
3	1.0000	0	0	0	0	0.4142	0.4142	0.4142	0.4142	1.0000	1.0000	1.0000	1.0000	1.2361	1.2361	1.2361	1.2361	1.2361	1.2361	1.2361	1.2361	1.8284	1.8284	1.8284
4	1.0000	0	0	0	0	0.4142	0.4142	0.4142	0.4142	1.0000	1.0000	1.0000	1.0000	1.2361	1.2361	1.2361	1.2361	1.2361	1.2361	1.2361	1.2361	1.8284	1.8284	1.8284
5	1.0000	0	0	0	0	0.4142	0.4142	0.4142	0.4142	1.0000	1.0000	1.0000	1.0000	1.2361	1.2361	1.2361	1.2361	1.2361	1.2361	1.2361	1.2361	1.8284	1.8284	1.8284
6	1.4142	0.4142	0.4142	0.4142	0.4142	0	0	0	0	0.5858	0.5858	0.5858	0.5858	0.8219	0.8219	0.8219	0.8219	0.8219	0.8219	0.8219	0.8219	1.4142	1.4142	1.4142
7	1.4142	0.4142	0.4142	0.4142	0.4142	0	0	0	0	0.5858	0.5858	0.5858	0.5858	0.8219	0.8219	0.8219	0.8219	0.8219	0.8219	0.8219	0.8219	1.4142	1.4142	1.4142
8	1.4142	0.4142	0.4142	0.4142	0.4142	0	0	0	0	0.5858	0.5858	0.5858	0.5858	0.8219	0.8219	0.8219	0.8219	0.8219	0.8219	0.8219	0.8219	1.4142	1.4142	1.4142
9	1.4142	0.4142	0.4142	0.4142	0.4142	0	0	0	0	0.5858	0.5858	0.5858	0.5858	0.8219	0.8219	0.8219	0.8219	0.8219	0.8219	0.8219	0.8219	1.4142	1.4142	1.4142
10	2.0000	1.0000	1.0000	1.0000	1.0000	0.5858	0.5858	0.5858	0.5858	0	0	0	0	0.2361	0.2361	0.2361	0.2361	0.2361	0.2361	0.2361	0.2361	0.8284	0.8284	0.8284
11	2.0000	1.0000	1.0000	1.0000	1.0000	0.5858	0.5858	0.5858	0.5858	0	0	0	0	0.2361	0.2361	0.2361	0.2361	0.2361	0.2361	0.2361	0.2361	0.8284	0.8284	0.8284
12	2.0000	1.0000	1.0000	1.0000	1.0000	0.5858	0.5858	0.5858	0.5858	0	0	0	0	0.2361	0.2361	0.2361	0.2361	0.2361	0.2361	0.2361	0.2361	0.8284	0.8284	0.8284
13	2.0000	1.0000	1.0000	1.0000	1.0000	0.5858	0.5858	0.5858	0.5858	0	0	0	0	0.2361	0.2361	0.2361	0.2361	0.2361	0.2361	0.2361	0.2361	0.8284	0.8284	0.8284
14	2.2361	1.2361	1.2361	1.2361	1.2361	0.8219	0.8219	0.8219	0.8219	0.2361	0.2361	0.2361	0.2361	0	0	0	0	0	0	0	0	0.5924	0.5924	0.5924
15	2.2361	1.2361	1.2361	1.2361	1.2361	0.8219	0.8219	0.8219	0.8219	0.2361	0.2361	0.2361	0.2361	0	0	0	0	0	0	0	0	0.5924	0.5924	0.5924
16	2.2361	1.2361	1.2361	1.2361	1.2361	0.8219	0.8219	0.8219	0.8219	0.2361	0.2361	0.2361	0.2361	0	0	0	0	0	0	0	0	0.5924	0.5924	0.5924
17	2.2361	1.2361	1.2361	1.2361	1.2361	0.8219	0.8219	0.8219	0.8219	0.2361	0.2361	0.2361	0.2361	0	0	0	0	0	0	0	0	0.5924	0.5924	0.5924
18	2.2361	1.2361	1.2361	1.2361	1.2361	0.8219	0.8219	0.8219	0.8219	0.2361	0.2361	0.2361	0.2361	0	0	0	0	0	0	0	0	0.5924	0.5924	0.5924
19	2.2361	1.2361	1.2361	1.2361	1.2361	0.8219	0.8219	0.8219	0.8219	0.2361	0.2361	0.2361	0.2361	0	0	0	0	0	0	0	0	0.5924	0.5924	0.5924
20	2.2361	1.2361	1.2361	1.2361	1.2361	0.8219	0.8219	0.8219	0.8219	0.2361	0.2361	0.2361	0.2361	0	0	0	0	0	0	0	0	0.5924	0.5924	0.5924
21	2.2361	1.2361	1.2361	1.2361	1.2361	0.8219	0.8219	0.8219	0.8219	0.2361	0.2361	0.2361	0.2361	0	0	0	0	0	0	0	0	0.5924	0.5924	0.5924
22	2.8284	1.8284	1.8284	1.8284	1.8284	1.4142	1.4142	1.4142	1.4142	0.8284	0.8284	0.8284	0.8284	0.5924	0.5924	0.5924	0.5924	0.5924	0.5924	0.5924	0.5924	0	0	0
23	2.8284	1.8284	1.8284	1.8284	1.8284	1.4142	1.4142	1.4142	1.4142	0.8284	0.8284	0.8284	0.8284	0.5924	0.5924	0.5924	0.5924	0.5924	0.5924	0.5924	0.5924	0	0	0
24	2.8284	1.8284	1.8284	1.8284	1.8284	1.4142	1.4142	1.4142	1.4142	0.8284	0.8284	0.8284	0.8284	0.5924	0.5924	0.5924	0.5924	0.5924	0.5924	0.5924	0.5924	0	0	0

Fig. S18. The values of the first 24×24 elements in the grid distance matrix $\bar{C}$ for sorted grid position.

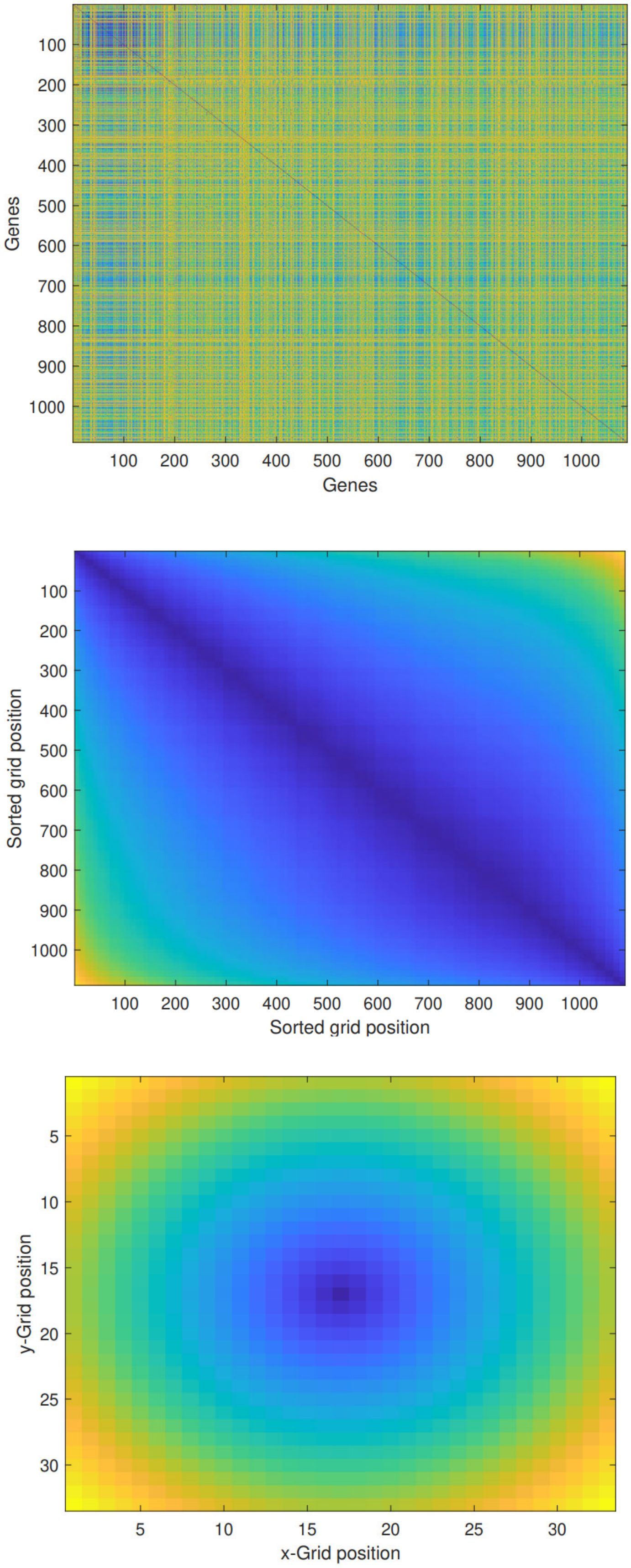

Fig. S19. The heatmaps of C=1-interaction matrix (1st row) and $\bar{C}$=grid distance matrix (2nd row) for the TM dataset. Grid distance matrix is computed from the mesh distance matrix shown in 3rd row. Grid positions are sorted before computing grid distance matrix here. From the grid distance matrix, it is seen that as the distance of a grid location from the center (1st row/column of $\bar{C}$-2nd row) increases, the distance value increases (see Fig. S18). Thus, when optimal transport is used to reduce the discrepancy between C and $\bar{C}$, it attempts to put highly interacting genes close to each other in the center and genes with low interaction to the periphery.

9. Analysis of genomap performance for selection of different number of HVGs and PCs

Table S6. Number of cells and genes of the datasets in genomap analysis

Dataset	Raw	Genomap analysis
Tabula Muris	19,791 genes and 54,865 cells	1,089 genes and 54,865 cells
Ischaemic sensitivity-lung	25,204 genes and 57,020 cells	2,704 genes and 57,020 cells
Ischaemic sensitivity-esophagus	24,245 genes and 87,947 cells	1,521 genes and 87,947 cells
Ischaemic sensitivity-spleen	22,612 genes and 94,257 cells	1,600 genes and 94,257 cells
T cell landscape	27,998 genes and 24,007 cells	1,089 genes and 24,007 cells
Transcriptome lineages of a proto-vertebrate	15,037 genes and 90,579 cells	729 genes and 90,579 cells
Retinal bipolar neurons	13,166 genes and 27,499 cells	1,089 genes and 27,499 cells

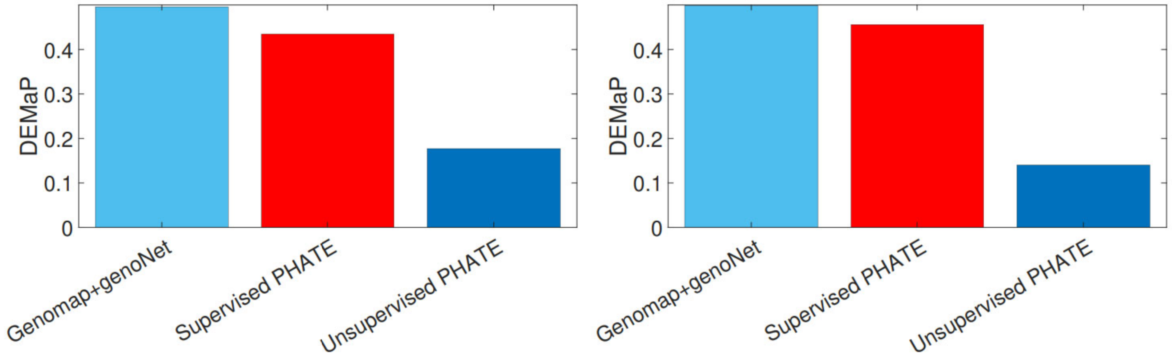

Fig. S20. DEMaP of different methods in trajectory analysis of proto-vertebrate data when 200 (left) and 300 (right) principal components are used. Source data are provided as a Source Data file.

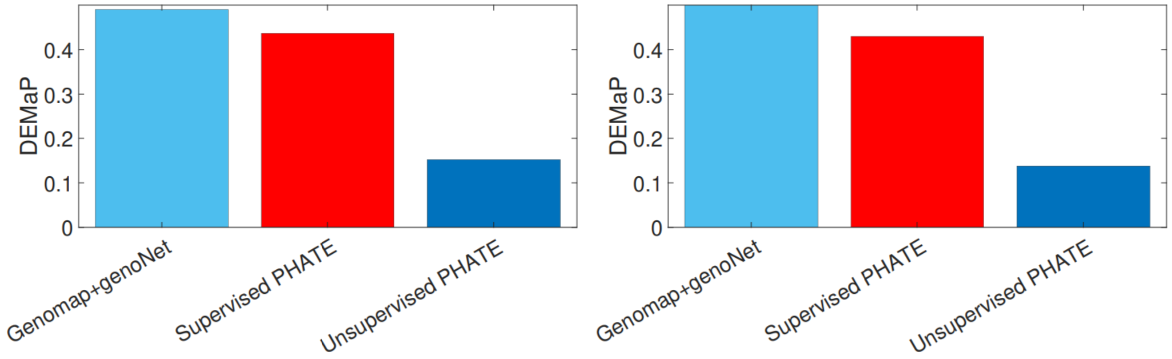

Fig. S21. DEMaP of different methods in trajectory analysis of proto-vertebrate data when 2000 (left) and 3000 (right) HVGs are used. Source data are provided as a Source Data file.

10. Color legends for cell classes

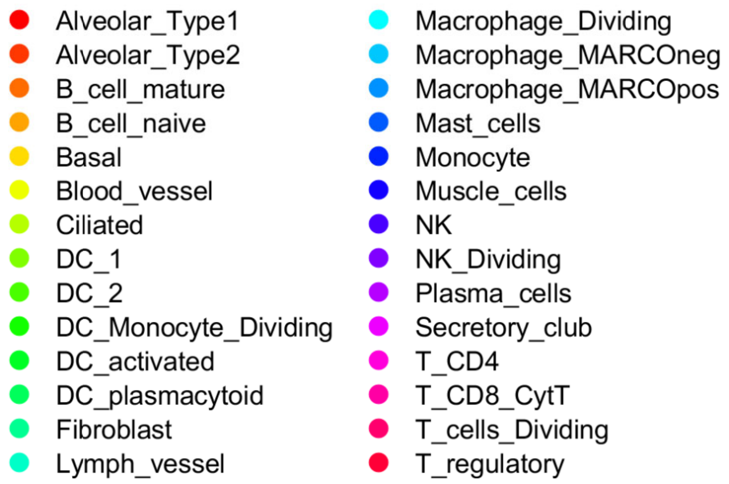

(a)

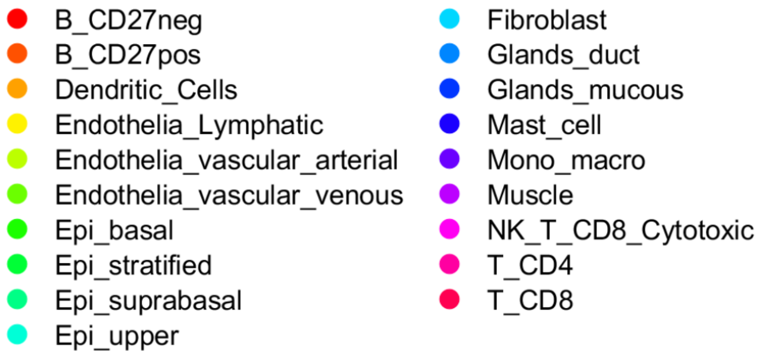

(b)

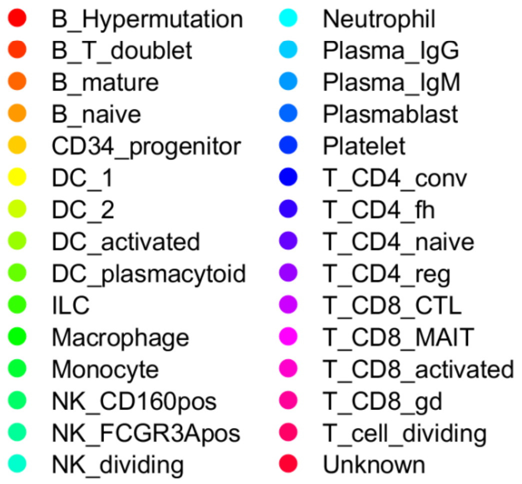

(c)

Fig. S22. Legends of ischemic sensitivity dataset (lung (a), esophagus (b) and spleen (c)).

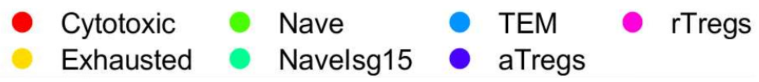

Fig. S23. Legends of T cell landscape dataset.

11. Reference

1. Lezon TR, Banavar JR, Cieplak M, Maritan A, Fedoroff NV (2006) Using the principle of entropy maximization to infer genetic interaction networks from gene expression patterns. *Proceedings of the National Academy of Sciences* 103(50):19033–19038.
2. Stein RR, Marks DS, Sander C (2015) Inferring Pairwise Interactions from Biological Data Using Maximum-Entropy Probability Models. *PLoS Comput Biol* 11(7):e1004182.
3. Razlighi QR, Kehtarnavaz N, Nosratinia A (2009) Computation of Image Spatial Entropy Using Quadrilateral Markov Random Field. *IEEE Transactions on Image Processing* 18(12):2629–2639.
4. Haralick RM, Shanmugam K, Dinstein I (1973) Textural Features for Image Classification. *IEEE Transactions on Systems, Man, and Cybernetics* SMC-3(6):610–621.
5. Shannon CE (1948) A mathematical theory of communication. *The Bell System Technical Journal* 27(3):379–423.
6. Dynkin E, et al. (1959) *Eleven Papers on Analysis, Probability and Topology*, American Mathematical Society Translations: Series 2. (American Mathematical Society) Vol. 12.
7. Tang H, Yu X, Liu R, Zeng T (2022) Vec2image: An explainable artificial intelligence model for the feature representation and classification of high-dimensional biological data by vector-to-image conversion. *Briefings in Bioinformatics* 23(2):bbab584.
