# Supplementary material for: Cartography of Genomic Interactions Enables Deep Analysis of Single-Cell Expression Data
Source: Nat Commun. 2023 Feb 8;14:679. doi: 10.1038/s41467-023-36383-6 (PMC9908983; doi:10.1038/s41467-023-36383-6)
Supplement: Supplementary file 3 — Reporting Summary [file 41467_2023_36383_MOESM3_ESM.pdf]

nature research

Corresponding author(s): Lei Xing
Last updated by author(s): Jan 23, 2023

Reporting Summary

Nature Research wishes to improve the reproducibility of the work that we publish. This form provides structure for consistency and transparency in reporting. For further information on Nature Research policies, see our Editorial Policies and the Editorial Policy Checklist.

Statistics

For all statistical analyses, confirm that the following items are present in the figure legend, table legend, main text, or Methods section.

n/a	Confirmed	
[ ]	[x]	The exact sample size (*n*) for each experimental group/condition, given as a discrete number and unit of measurement
[ ]	[x]	A statement on whether measurements were taken from distinct samples or whether the same sample was measured repeatedly
[x]	[ ]	The statistical test(s) used AND whether they are one- or two-sided<br>*Only common tests should be described solely by name; describe more complex techniques in the Methods section.*
[x]	[ ]	A description of all covariates tested
[ ]	[x]	A description of any assumptions or corrections, such as tests of normality and adjustment for multiple comparisons
[x]	[ ]	A full description of the statistical parameters including central tendency (e.g. means) or other basic estimates (e.g. regression coefficient) AND variation (e.g. standard deviation) or associated estimates of uncertainty (e.g. confidence intervals)
[x]	[ ]	For null hypothesis testing, the test statistic (e.g. *F*, *t*, *r*) with confidence intervals, effect sizes, degrees of freedom and *P* value noted<br>*Give P values as exact values whenever suitable.*
[x]	[ ]	For Bayesian analysis, information on the choice of priors and Markov chain Monte Carlo settings
[x]	[ ]	For hierarchical and complex designs, identification of the appropriate level for tests and full reporting of outcomes
[x]	[ ]	Estimates of effect sizes (e.g. Cohen's *d*, Pearson's *r*), indicating how they were calculated

Our web collection on statistics for biologists contains articles on many of the points above.

Software and code

Policy information about availability of computer code

Data collection	All public datasets used are reported in the manuscript. No special software was used to collect the data. For creating Fig. 4, we used the Python codes from https://github.com/elo073/TissStab provided by the original authors.
Data analysis	The custom codes were written both in Python 3.8 and Matlab 2020b. Data analyses were performed in both languages.

For manuscripts utilizing custom algorithms or software that are central to the research but not yet described in published literature, software must be made available to editors and reviewers. We strongly encourage code deposition in a community repository (e.g. GitHub). See the Nature Research guidelines for submitting code & software for further information.

Data

Policy information about availability of data

All manuscripts must include a data availability statement. This statement should provide the following information, where applicable:

- Accession codes, unique identifiers, or web links for publicly available datasets
- A list of figures that have associated raw data
- A description of any restrictions on data availability

The accession numbers for the datasets used in the paper are: Baron (GSE84133), Muraro (GSE85241), Segerstolpe (E-MTAB-5061), Xin (GSE81608), Wang (GSE83139), ischaemic sensitivity (PRJEB31843), TM (GSE109774). T cell landscape, proto-vertebrate transcriptomics, and retinal bipolar neuron datasets were downloaded from single cell portal of Broad institute with study number SCP490, SCP454, and SCP3, respectively. Note that the user needs to register to the single cell portal of Broad Institute with an email and a password. He/she can then login into the portal with the email and password and download the datasets.

Field-specific reporting

Please select the one below that is the best fit for your research. If you are not sure, read the appropriate sections before making your selection.

[x] Life sciences [ ] Behavioural & social sciences [ ] Ecological, evolutionary & environmental sciences

For a reference copy of the document with all sections, see nature.com/documents/nr-reporting-summary-flat.pdf

Life sciences study design

All studies must disclose on these points even when the disclosure is negative.

Sample size	The datasets were acquired from established databases. All the samples from the datasets were analyzed and no subsampling was performed. The sample size varied from 24,007 cells for T cell landscape dataset to 94,257 cells for ischemic sensitivity-spleen dataset. The superiority of genomap in analyzing the expression values for this large range of sample size proves its wide applicability. The sample size was enough because of small variability in performance of genomap.
Data exclusions	No data were excluded from the analyses.
Replication	All experiments were conducted in a manner that produced clean replicates. The experiments regarding application of genomap were replicated 20 times on each dataset used in the study. All attempts at replication were successful.
Randomization	No data allocation was performed randomly. No covariate was controlled. As the recovery of gene expression does not require any prior knowledge about the covariates or experimental groups, such information was not relevant to our method or study.
Blinding	There was no blinding. The analyses performed do not involve evaluation of any subjective matters.

Reporting for specific materials, systems and methods

We require information from authors about some types of materials, experimental systems and methods used in many studies. Here, indicate whether each material, system or method listed is relevant to your study. If you are not sure if a list item applies to your research, read the appropriate section before selecting a response.

Materials & experimental systems

n/a	Involved in the study
[x]	[ ] Antibodies
[x]	[ ] Eukaryotic cell lines
[x]	[ ] Palaeontology and archaeology
[x]	[ ] Animals and other organisms
[x]	[ ] Human research participants
[x]	[ ] Clinical data
[x]	[ ] Dual use research of concern

Methods

n/a	Involved in the study
[x]	[ ] ChIP-seq
[x]	[ ] Flow cytometry
[x]	[ ] MRI-based neuroimaging
